# Rationale and Methods for a Randomized Controlled Trial of a Dyadic, Web-Based, Weight Loss Intervention among Cancer Survivors and Partners: The DUET Study

**DOI:** 10.3390/nu13103472

**Published:** 2021-09-29

**Authors:** Dorothy W. Pekmezi, Tracy E. Crane, Robert A. Oster, Laura Q. Rogers, Teri Hoenemeyer, David Farrell, William W. Cole, Kathleen Wolin, Hoda Badr, Wendy Demark-Wahnefried

**Affiliations:** 1 Department of Health Behavior, University of Alabama at Birmingham (UAB), Birmingham, AL 35294, USA; 2 O’Neal Comprehensive Cancer Center at University of Alabama at Birmingham (UAB), Birmingham, AL 35294, USA; roster@uabmc.edu (R.A.O.); lqrogers@uabmc.edu (L.Q.R.); demark@uab.edu (W.D.-W.); 3 Sylvester Comprehensive Cancer Center, Miami, FL 33136, USA; tecrane@med.miami.edu; 4 Division of Preventive Medicine, Department of Medicine, Birmingham, AL 35294, USA; 5 Department of Nutrition Sciences, University of Alabama at Birmingham (UAB), Birmingham, AL 35294, USA; tgw318@uab.edu (T.H.); colew14@uab.edu (W.W.C.); 6 People Designs, Inc., Durham, NC 27705, USA; dfarrell@peopledesigns.com; 7 Coeus Health, LLC, Chicago, IL 60614, USA; kate@drkatewolin.com; 8 Department of Medicine, Epidemiology and Population Sciences, Baylor College of Medicine, Houston, TX 77030, USA; hoda.badr@bcm.edu

**Keywords:** diet, weight loss, exercise, physical activity, lifestyle, cancer survivors, Internet, dyads

## Abstract

Scalable, effective interventions are needed to address poor diet, insufficient physical activity, and obesity amongst rising numbers of cancer survivors. Interventions targeting survivors and their friends and family may promote both tertiary and primary prevention. The design, rationale, and enrollment of an ongoing randomized controlled trial (RCT) (NCT04132219) to test a web-based lifestyle intervention for cancer survivors and their supportive partners are described, along with the characteristics of the sample recruited. This two-arm, single-blinded RCT randomly assigns 56 dyads (cancer survivor and partner, both with obesity, poor diets, and physical inactivity) to the six-month DUET intervention vs. wait-list control. Intervention delivery and assessment are remotely performed with 0–6 month, between-arm tests comparing body weight status (primary outcome), and secondary outcomes (waist circumference, health indices, and biomarkers of glucose homeostasis, lipid regulation and inflammation). Despite COVID-19, targeted accrual was achieved within 9 months. Not having Internet access was a rare exclusion (<2%). Inability to identify a support partner precluded enrollment of 42% of interested/eligible survivors. The enrolled sample is diverse: ages 23–81 and 38% racial/ethnic minorities. Results support the accessibility and appeal of web-based lifestyle interventions for cancer survivors, though some cancer survivors struggled to enlist support partners and may require alternative strategies.

## 1. Introduction

Given the high number of cancer survivors in the United States (over 16.9 million in 2019 [1]) due to improvements in early detection and treatment, new challenges emerge in terms of preventing second malignancies and common comorbidities and promoting quality of life (QoL) and healthy aging among survivors. Healthy diet, weight management, and physical activity can enhance the quality (and quantity) of life for cancer survivors and reduce their risk for developing secondary cancers; however, few cancer survivors meet the World Cancer Research Fund (WCRF) and American Institute of Cancer Research (AICR) recommendations for diet and physical activity [2]. Moreover, recent National Health Interview Survey (NHIS) data indicate that 34% of adult cancer survivors report no leisure time physical activity and 33% have obesity [3], which clearly demonstrates the need for effective, scalable lifestyle interventions in this population.

Past studies have reported changes in diet, physical activity and/or weight loss among cancer survivors, with most interventions delivered face-to-face or via print and/or telephone [4]. For example, in the RENEW study, tailored mailed print materials and telephone counseling produced significant improvements in diet quality, physical activity, and body mass index at 12 months among 641 older survivors of breast, prostate, and colorectal cancer with overweight and obesity, which were sustained at two-year follow-up [5,6]. However, higher reach, more cost effective, technology-supported strategies might be required to address a public health problem of this magnitude.

Website intervention delivery requires less staff time and training than face-to-face and telephone-based approaches; furthermore, the incremental delivery costs per participant is minimal. Promising results were reported in a recent review of web-based lifestyle interventions for cancer survivors [7]; however, most of the programs were only 6–12 weeks in duration and did not assess long-term behavior change. As cancer survivors experience unique health concerns, more support and engagement may be required to achieve long-term maintenance of lifestyle changes.

Enrolling cancer survivors with support partners could lead to more sustainable gains in health behaviors. A dyad-based approach, in which 68 inactive breast cancer survivors with overweight or obesity partnered with their adult daughters who had similar characteristics, showed promise in the daughters and mothers (DAMES) study (n = 136) [8]. The dyads who received the tailored, self-help, print materials experienced significant improvements in body mass index (BMI), weight, waist circumference (WC), and physical activity at 12 months, compared to control arm dyads who received standard, publicly available brochures on diet, exercise, and weight status. Building on this research, the current study seeks to combine both the advantages of web-based platforms and partner support by developing and testing the first (to our knowledge) dyad- and web-based lifestyle intervention for cancer survivors.

The current paper describes the rationale, design, and recruited sample for an ongoing efficacy trial of DUET (daughters, dudes, mothers, and others together), a six-month web-based lifestyle intervention to promote weight loss among cancer survivors with overweight or obesity and their chosen supportive partners. The central hypothesis is that cancer survivor and support partner dyads that are assigned to the web-based intervention will lose significantly more weight at six months than dyads in the wait-list control. We also expect that the intervention will result in more favorable changes in other measures of adiposity (e.g., BMI and WC), diet quality, physical activity, QoL, and physical functioning and performance, as well as related biomarkers (e.g., insulin, glucose, total and high-density lipoprotein (HDL) cholesterol, triglycerides, leptin, adiponectin, interleukin 6(IL6), c-reactive protein (CRP), and tumor necrosis factor alpha).

## 2. Materials and Methods

### 2.1. Overall Design

DUET is a single-blinded, 2-arm randomized controlled trial (RCT) that will test a 6-month, web-based lifestyle intervention against a wait-list control among 56 dyads. Each dyad is comprised of a survivor of an obesity-related early-stage cancer and their supportive partner, both of whom have obesity or overweight, are insufficiently active, and consume suboptimal diets. The main outcome (body weight) is assessed at baseline and 6 months, along with several other secondary outcomes. This trial was approved by the University of Alabama at Birmingham (UAB) Institutional Review Board (300003882/Approval date: 10-28-2019) and registered with ClinicalTrials.gov (NCT04132219).

### 2.2. Participant Recruitment and Eligibility Screening

A two-step recruitment process was undertaken whereby initial enrollment efforts targeted cancer survivors, and once interest and eligibility were established, efforts were directed towards enrolling appropriate partners. Survivors of obesity-related cancers with 5-year cancer-free survival rates of at least 70% (i.e., localized renal cancer, and loco-regional ovarian, colorectal, prostatic, endometrial, and female breast cancers) [9]. Adult survivors of these cancers were identified through the UAB Cancer Registry, as well as through a wait-list of individuals who had previously expressed interest in lifestyle interventions and provided their contact email address or telephone numbers. Letters of invitation were posted to registry-ascertained cases, and telephone calls or email messages were placed to individuals on the wait-list. The Love Research Army (https://drsusanloveresearch.org/love-research-army, accessed on 27 September 2021) also initiated a series of email “blasts” to its members, and a recruitment website was established (https://duet4health.org, accessed on 27 September 2021). Finally, individuals not meeting eligibility criteria or disinterested in other ongoing cancer survivorship studies were apprised of DUET.

Study staff provided telephone follow-up on recruitment mailings and contacts by placing up to six calls at various days and times. The study was explained and interested survivors were screened for eligibility. Inclusion and exclusion criteria were established to target survivors who were most in need and who could best benefit from a web-based diet and exercise intervention. Inclusion criteria were as follows: (1) BMI > 25 kg/m^2^ [10]; (2) vegetable and fruit intake <2.5 cups/day; (3) moderate-to-vigorous physical activity (MVPA) <150 min/week [11]; (4) completion of primary cancer treatment; (5) English speaking and writing; (6) educational attainment of 5th grade or higher; and (7) daily use of the Internet and mobile phone access. Exclusion criteria were few and limited to those who were already adhering to modified diets or enrolled in an exercise program, residing in assisted or skilled nursing facilities, or recently advised by their physician to limit physical activity and/or having health issues that might make participation in an unsupervised weight loss intervention unsafe (e.g., pregnancy, severe orthopedic conditions, end-stage renal disease, metastatic cancer or other cancers with poorer survival, paralysis, dementia, blindness, unstable angina, untreated stage 3 hypertension, recent history of heart attack, congestive heart failure or pulmonary conditions that required oxygen or hospitalization within 6 months) [12]. Once initial eligibility was established and cancer case status was verified by treating physicians of any self-referrals, the survivor was asked to identify a local support partner (preferably within a 10-min drive) and have them contact the research team for screening. Supportive partners were required to meet all inclusion/exclusion criteria, except for being a cancer survivor.

### 2.3. Study Protocol

The research team provided study overviews for eligible dyads and answered their questions via conference calls. Informed consent was obtained from all participants involved in the study and signed electronically (Adobe Sign^®^, San Jose, CA, USA). Participants completed baseline assessments and dyads were randomly and evenly assigned to study arms (DUET intervention or wait-list control) using a permuted block design (block size = 4). Participants complete assessments again at 6 months and then wait-list control dyads receive the DUET intervention.

### 2.4. DUET Intervention

The DUET web-based intervention was adapted from the previously mentioned tailored, mail-delivered dyadic DAMES intervention [8] and then expanded to meet the needs of a broader range of cancer survivors and support partners (i.e., not limited to post-menopausal breast cancer survivors and their biological daughters). Like DAMES, DUET was theoretically grounded and primarily based on the social cognitive theory (SCT) [13]; which posits that participation in health behaviors is determined by individual factors (e.g., self-efficacy, or confidence in the ability to exert control over one’s own behavior) and the social and physical environment (e.g., barriers, social support from friends and family). The DUET intervention targets key SCT constructs by providing participants with resources (i.e., Fitbits and Aria Scales) to track diet, exercise, and weight, and provides guidance on setting incremental goals. Such strategies build upon small successes with lifestyle change and thereby enhance self-efficacy. The DUET weekly sessions also directly address weight loss barriers that are common for cancer survivors, such as fatigue and stress, as well as barriers common across populations, such as time constraints, to address the needs of both partners.

To further bolster dyadic interactions to enhance social support, concepts from interdependence theory and the theory of communal coping were incorporated [14]. DUET emphasizes relational factors such as joint problem solving, commitment to relationship quality and upholding mutual goals to promote adoption and maintenance of health behaviors. Moreover, dyads receive guidance on supporting their partners (e.g., how to ask for and provide help). In total, the DUET intervention draws upon 38 of the 40 behavioral change techniques that are categorized by the CALO-RE taxonomy purported by Michie and colleagues to promote adherence to healthful diet and physical activity patterns [15]. The two exceptions to the taxonomy are formal motivational interviewing (MI) and fear arousal. Although some MI elements were incorporated into the website design and information on cancer risk and recurrence and comorbidity are presented, emotionally evocative images were purposely avoided given the already high levels of anxiety related to such outcomes among cancer survivors and their loved ones [16].

The website includes the following sections: My Profile, Healthy Weight, Healthy Eating, Exercise, Weekly Sessions, Tools, News You Can Use, and Team Support. All users are given instructions on using the website features and encouraged to call the research team with any problems or questions. Once logged in, participants can update personal information (gender, age, height, weight, diet, physical activity, and cancer history) in the My Profile feature and then access tailored content in the Healthy Weight, Healthy Eating, and Exercise sections. Participants pursue and track their weight, diet, and exercise using study-provided equipment (Fitbit^®^ Aria 2 digital scales and Inspire fitness trackers (San Francisco, CA, USA), Portion Doctor^®^ tableware (Portion Health Products, St. Augustine Beach, FL, USA) and exercise bands (Theraband Academy, Akron, OH, USA)). Dyads also are encouraged to work as a team and use the commercially available MyFitnessPal (https://www.myfitnesspal.com/, accessed on 27 September 2021) app to set goals and view progress; log-ins and data are tracked to assess adherence. Participants are cued via text messages to complete 24 weekly interactive diet and exercise modules in the Sessions section; sessions range from 10–20 min. See Table 1 for session topics.

The Tools section includes tracking forms, online calculators, planning guides, tip sheets, and other healthy eating and exercise resources. Summarized updates on recent findings from salient research on diet, exercise, and/or weight loss for cancer prevention and control is provided in the News feature. The Team Support page offers practical tips on how dyads can support each other to promote lifestyle change (e.g., active listening). Regular website usage is encouraged via text messages (three per week) and tracked to assess intervention adherence. Short Message System (SMS) Text Messages also are a key component of the DUET intervention. After an initial welcome message, text messages are delivered at a frequency of three per week over the course of the intervention for a total of 72 messages for intervention.

Intervention adherence is evaluated using a variety of means. Completion of sessions, as well as website logins/duration and text message receipts/responses, are all tracked. Moreover, the dyad provides permission for the research office to access secure Fitbit wireless API generated by the Inspire tracker and Aria scale. These data are downloaded by study staff at intervention completion and stored on the study server by ID number until analysis.

### 2.5. Assessments

Baseline and 6-month follow-up assessments are largely identical except that some demographic and health characteristics that are likely to be time invariant for an adult sample over the study period (e.g., race/ethnicity, marital and educational status, height) are self-reported only at enrollment. Originally designed to include home-based assessments, the DUET protocol was modified prior to recruitment to virtual assessments via Zoom^®^ (San Jose, CA, USA) in order to continue research activities during the COVID-19 pandemic. Assessors were trained and evaluated for accuracy prior to initiation; measures were evaluated for reliability, as well as validity with those collected in-person data that are featured in a separate report [17]. All Zoom^®^ sessions are recorded to increase accuracy for timed performance testing to reduce discrepancies resulting from variable transmission of sight and sound and allow for periodic quality assurance evaluations among assessors. Once assessors review these files, time the tests, log the data, and quality assurance tests are completed, the recordings are deleted. Virtual assessments are scheduled during times when both dyad members can participate and occur in tandem with one member of the dyad undergoing the assessment first and the other recording the encounter on Zoom^®^, and then vice versa. Dyads are asked to prepare by viewing videos on performance testing (https://youtu.be/lbxctNuOgLk, accessed on 27 September 2021) and dried blood spot (DBS) collection (https://youtu.be/lBPLS4PoHv4, accessed on 27 September 2021).

Supplies also are sent to the home of the dyad member in which assessments will be performed. Mailed materials include an 8′ length of cord and two stickers (to mark the distance for the 8′ walk and up-and-go performance tests), two orange soccer cones (to enhance virtual visualization for walk testing), and a 36″ vinyl tape measure and two stickers (to measure and guide step height for 2-min step tests). The mailing also includes duplicate supplies to cover the assessment needs of each dyad member: (1) programmed Actigraphs (Walton Beach, FL, USA) with activity/sleep logs; (2) DBS kits (903TM Protein Saver Card, 2 lancets, 2 non-stick gauze pads, 2 small adhesive bandages, 2 alcohol prep wipes, 1–5 × 3″ foil biohazard envelop with desiccant) to self-collect fasting blood samples (12 h or more); and (3) Two ribbons (4–1″ × 55″) and a felt-tip marker (to perform repeated measures of WC). A digital scale is sent if participants do not have one. In addition to the virtual assessment and bio-specimen collection, each dyad member completes an on-line survey and a 2-day dietary recall conducted by telephone at each time point. Details of specific measures are provided in Table 2.

As indicated and in addition to comorbidity, other potential moderators of the intervention’s effect on weight change, such as demographic factors, distance separating the dyad members (and dyad cohabitation vs. not), smoking status [30], and risk for depression (as measured by the PROMIS Cancer-Related Item Bank) will be explored [31]. Potential mediators of effect also will be studied and include specific SCT constructs directly targeted by the intervention (e.g., self-efficacy, social support, and barriers). Self-efficacy will be measured with a 20-item instrument (α = 0.70–0.88) for dietary weight management [32] and the 6-item Lifestyle Efficacy scale (α = 0.95) [33]. Social support for these lifestyle changes will be assessed using validated 5-point scales with acceptable test-retest reliabilities (r = 0.55–0.86) and internal consistencies (α = 0.61–0.91) [34]. Barriers will be captured using a list of 36 common barriers to a diet with reduced fat and sugar, and increased fruits and vegetable intake, whole grains, and exercise (cost, availability, time, etc.) [35,36,37,38].

Upon completion of the intervention both dyad members undergo separate telephone debriefings on the acceptability and satisfaction of the various intervention components (e.g., website, equipment, text messages) and their suggestions for improvement are solicited.

As with any lifestyle intervention trial conducted in a high-risk patient population, especially one that is home-based and unsupervised, adverse events are a key concern. Thus, changes in health status of both study arms are systematically ascertained at study midpoint (3 months), in addition to 6-month follow-up. Any hospitalizations are logged, and admittance to the hospital resulting in an overnight stay, as well as events that are permanently disabling or life threatening are categorized as “serious” with attribution of the intervention explored further. Furthermore, all study participants are encouraged to call a toll-free study number to report any adverse events that occur between assessments.

### 2.6. Statistical Power

This 2-arm RCT formally tests for differences in the loss of body weight from baseline to 6-months that occurs among 56 dyads (with each dyad comprised of a survivor and a partner) who are randomized to two study arms. All other analyses and tests are exploratory. Power calculations are based on the following assumptions: (1) the retention rate will be identical to the DAMES trial (i.e., 90%), which is conservative as the duration for DUET is only 6 months instead of 12 months; (2) the eHealth intervention will promote weight losses of ~3.46 kg during the 6-month study period (similar to those observed in the Healthy Moves tailored-print, web-based, spousal support intervention—see companion article by Carmack et al. in this Nutrients edition [39]), whereas the control arm will be weight stable—a conservative estimate, since the average American gains 0.5–1 kg/year [40]); and (3) two-sided tests at an alpha level of 0.05 will be used. Given these assumptions, along with those of a two-sided two-group *t*-test, a standard deviation of 4.6 kg (from Healthy Moves [39]), and a sample size of 25 dyads per arm (allowing for 90% retention of the initial 28 dyads per arm), there is at least 80% power to detect differences in weight loss of −3.72 kg or greater between the two arms.

### 2.7. Data Analyses

The primary analysis will be performed on an intent-to-treat basis using baseline to 6-month data. Arm differences in weight loss will be assessed using a mixed linear model which accounts for the covariance between the dyad members. Specifically, mixed models repeated measures analyses will be used to test differences between arms, the two time points, and the potential interaction between arm and time point simultaneously. An appropriate structure for the covariance matrix (e.g., unstructured) will be selected using the final data. Clinical and demographic covariates of interest will be included in these models. The Tukey-Kramer multiple comparisons test will be used to determine which pairs of means are significantly different. Overall cross-sectional comparisons of continuous variable at baseline will be performed using the two-group *t*-test to determine if there are any differences remaining between the arms after randomization. For categorical variables, comparisons between arms will be performed using the two-group chi-square test (or Fisher’s exact test if the assumptions for the chi-square test are not tenable). The strength of the relationship between pairs of variables will be examined using Pearson (or Spearman, if needed) correlation analyses. Distributions of continuous study variables will be examined using stem-and-leaf, box, and normal probability plots and the Kolmogorov-Smirnov test; any of these variables that deviate from a normal distribution will be transformed prior to analysis or will be analyzed using non-parametric methods such as the Wilcoxon rank-sum test.

Statistical tests will use an alpha level of 0.05 and will be two-sided. SAS software (version 9.4 or later; SAS Institute, Inc., Cary, NC, USA) will be used to perform all statistical analyses. Analyses of secondary outcomes will occur similarly, though analyses are exploratory, and as such will not be controlled for multiple testing. To identify predictor variables associated with program efficacy, e.g., social support (type, amount), self-efficacy, and risk of depression, logistic regression analyses will be used. Odds ratios, along with their corresponding two-sided 95% confidence intervals, will be obtained for all variables included in these models.

For our initial statistical analyses that focus on determining potential demographic and cancer-type differences between enrolled DUET cancer survivors and partners, between cancer survivors who express interest in the DUET intervention versus those who refuse or are unresponsive, and those who enroll in the RCT versus those not enrolled, two-group t-tests are performed for continuous variables such as age, and chi-square tests are performed for categorical variables, such as gender, race/ethnicity, residence in a rural- or urban-classified county, and cancer-type. These analyses are now complete, and the results are presented in the next section—findings that are integral in assessing program interest and to appropriately generalize the main outcomes of this trial upon its completion.

## 3. Results

Recruitment for DUET spanned 8 October 2020 to 2 July 2021, and despite substantial overlap with the COVID-19 pandemic, met its accrual target of 56 partnered dyads within a 9-month period. To date, there have been no drop-outs; however, the trial is still in the field with completion of data collection anticipated within the next five months. Laboratory and statistical analyses will occur over the subsequent 6-month period.

**Figure 1:**
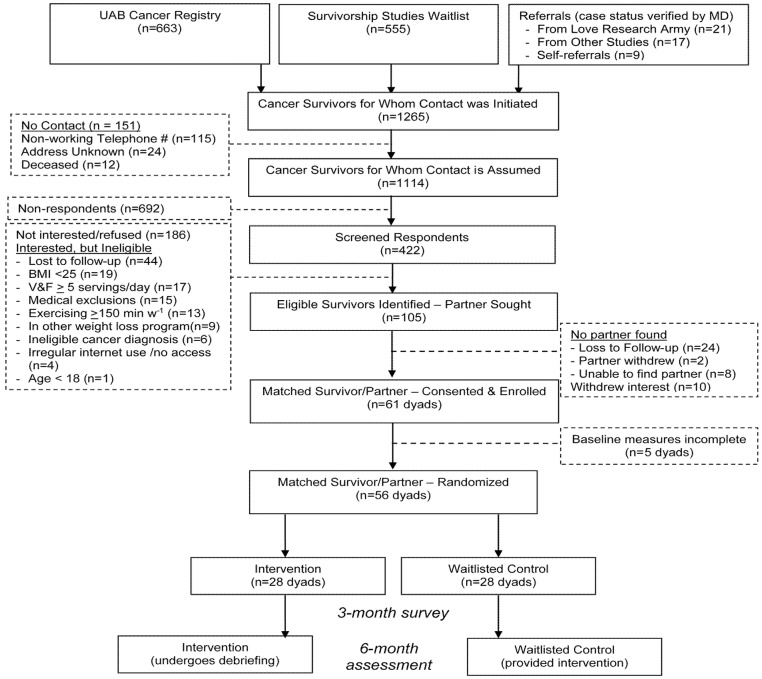
DUET study flow diagram that focuses on the enrollment trajectory.

Figure 1 details the study trajectory from self-referral or registry/wait-list ascertainment to randomization. Data suggest that for intervention RCTs like DUET, roughly 23 cancer survivors require contact for every participant enrolled. Granted, this number could be reduced to less than 20 if contact data in registries were current, but roughly 12% of cases were found to be deceased or had telephone and/or address information that was obsolete. Of those for whom contact is assumed, roughly 21% express interest in participating in the trial, but over one-third (37%) screen-out on various eligibility criteria with the leading causes for exclusion being normal or underweight status, already adhering to a healthful diet or regular exercise, or medical exclusions.

Of note, irregular computer use, or lack of Internet access was a rarely reported occurrence, with less than 2% being screened-out on this criterion. Moreover, while a substantial number of cancer survivors initially expressed interest, almost 20% were lost to follow-up afterward. This substantial loss to follow-up also occurred once enrollment became focused on partners; here loss to follow-up accounted for 28% of individuals identified either prior to consent or the baseline appointment. Additionally, the identification of a partner appeared to be a barrier since 42% of interested survivors were unable to enlist one.

DUET enrolled participants in Alabama, Illinois, Mississippi, North Carolina, and Tennessee. Table 3 provides the characteristics of the DUET cohort. The sample of survivors is comprised largely of individuals diagnosed with female breast cancer, though early-stage kidney, prostate, endometrial and ovarian cancer also are represented. Interestingly, 13% of supportive partners also reported cancer histories, again with most of these diagnoses being female breast cancer.

By and large, these are long-term cancer survivors more than five years (M = 67.5 months) out from diagnoses. Given the high representation of breast cancer (80%), it is unsurprising that most participants are female (86%). The age range is broad (i.e., 23–81 years) with a mean age of 58.4 years, and while most participants are employed (55%), one-third are retired. In addition, most (85%) acknowledged at least some college education, with a substantial proportion (43%) reporting annual incomes of at least $50,000 or refusing to answer this question. Minorities comprise almost 40% of the sample, with non-Hispanic Blacks (NHB) having the highest representation; however, very few participants are rural. While most dyads resided separately, over 40% cohabitated with their supportive partners (all of cohabitating partners were in spousal relationships). Both survivors and their supportive partners had average BMI’s falling in the range of Class I obesity (M BMI = 32).

Tests that compared the enrolled DUET cancer survivors (*n* = 56) to partners (*n* = 56) found significant differences for cancer diagnosis (*p* < 0.001), where the proportion of survivors with cancer (100%) was greater than the proportion of partners with cancer (13%), and for gender (*p* = 0.025), where the proportion of female survivors (86%) was greater than the proportion of female partners (68%).

Tests that compared the sample who responded with interest (*n* = 236) and ultimately enrolled in DUET (*n* = 56) to the larger pool who were unresponsive (*n* = 1029) and unenrolled (*n* = 1209) found no significant differences by rural (versus urban) residence or race/ethnicity (*p* > 0.10 for both); however, there were significantly higher response rates among survivors of breast cancer (and hence, females), as well as those who are younger (*p* < 0.05 for both). Though after screening, the only clear difference between enrolled participants versus the potential pool was the proportion of breast cancer survivors, which was significantly higher among enrollees (*p* < 0.0001).

## 4. Discussion

To our knowledge, DUET is the first interactive web-based intervention aimed at improving body weight status, dietary intake, and physical activity among high-need survivors of obesity-related cancers and their supportive partners. As such, it represents a program that not only addresses the tertiary prevention needs of cancer survivors, but also serves to promote primary prevention among their friends and family members—a substantial proportion of whom are at higher risk due to common risk factors. In addition, DUET is unique from the perspective of capturing outcome data strictly using remote methodologies. Thus, it is among a new generation of trials in which the intervention is delivered, and the outcomes are assessed exclusively via remote means. The fact that we were able to meet our accrual target for this logistically challenging trial involving a 2-step process among both cancer survivors and partners within nine months, and during a pandemic when other cancer prevention and control trials are struggling (i.e., Unger et al. reports a decrease of 54% in enrollment during the same period [41]), is testimony to the fact that web-based approaches have appeal.

Remote delivered and assessed trials also have the ability to recruit participants broadly and thereby potentially increase the generalizability of findings. As such, DUET was able to engage participants residing in a broad swath of America, from Illinois to Alabama. Moreover, it attracted cancer survivors and supportive partners across a vast age range that extended from individuals in their second through eighth decades of life. It also enrolled a racial and ethnically diverse cohort, as supported by a minority accrual of almost 40%, thereby surpassing U.S. Bureau of Census statistics that suggest 31% for the mean age group of this sample (i.e., 58–59 years) [42]. Indeed, web-based trials remove several barriers that are commonly reported for both older and minority populations, such as transportation and time away from family or occupational commitments [43]. Furthermore, the concern that cancer survivors and their partners would not have Internet access nor adequate computer skills was unfounded based on our data that less than 2% of survivors screened-out on this criterion and there has been good uptake of the intervention to date. Albeit this percentage is far lower than the 18% computer-related exclusion that was recently reported by van der Hout and colleagues for a web-based supportive care intervention across a mixed sample of cancer survivors; however, recruitment for their Oncokompas trial occurred in 2016–2017 [44]. Given estimates indicating that there are on average 640,000 new users of the Internet each day globally (with sharp increases during the pandemic) [45], the concern that cancer survivors may be unreachable through web-based programs appears to be diminishing rapidly.

A much greater barrier to accrual was the identification of a partner in order to participate in this dyadic-based intervention. Forty-two percent of interested and eligible cancer survivors were unable to engage a supportive partner. While this proportion is lower than the 48% suggested by the DAMES trial, there was an expectation that expanding the criteria for a partner beyond just a biological child to include other family members, spouses, friends, and neighbors, would yield a far better response rate—it did not. Therefore, this is a key concern for dyadic-based interventions in the future, at least for those that are aimed at improving diet quality, physical activity and weight status among cancer survivors and their circles of friends and family members. That being said, the magnitude of change possible for dyadic interventions needs to be weighed against these logistical considerations. The fact that both the DAMES and Healthy Moves trials resulted significant improvements in vegetable and fruit consumption and/or weight loss with modest-sized samples suggests that although dyadic interventions are challenging, they still may be worth the effort [8,39]. The results for DUET will add substantially to this small body of research.

Of note, the considerable representation of dyad spouses within the DUET cohort and the relative ease with which the Healthy Moves cohort was assembled, suggests that the spousal relationship is perhaps the most fruitful to capitalize upon and engage potential participants [39]. Because the DUET sample is relatively evenly divided between survivor-spouse dyads versus dyads comprised of survivors and others, it will be one of the first (if not the first) to compare changes that occur in health behaviors and outcomes changes in these two different subgroups. While our study is likely to be underpowered in detecting significant differences, the descriptive data that result still will be helpful in supporting frameworks such as that proposed by Monterrosa et al. that identify and categorize the several different influences on food choices on various social and environmental levels [46]. Given that the spousal relationship and its inherent cohabitation affect food procurement and preparation, and other more far-reaching domains, we anticipate that intervention effects may be accentuated in this subgroup.

As stated, the outcomes of the DUET RCT are anticipated within the next calendar year. Given its potential to break new ground in the fairly small (yet growing) areas of dyadic interventions, as well as remote intervention delivery and evaluation, results should be of interest to researchers not only involved in cancer control, but also interventionists who implement diet and exercise interventions within a variety of patient populations and to prevent a multitude of chronic diseases. Strengths of DUET include its randomized controlled design, theoretically-grounded intervention, and attention to fidelity. As with all studies, DUET has limitations which include a significant overrepresentation of breast cancer survivors (many of whom are upper-socioeconomic), and few dyads with rural residence. These are common limitations that have been reported by other research teams [47,48,49]. To address these concerns, future studies might consider preferentially accruing from support groups that focus on other types of cancer and community cancer centers in smaller towns (rather than major tertiary oncologic care centers). Finally, and potentially as a last resort, future studies may need to stratify accrual by cancer type, socio-economic factors and/or rural/urban residence to assure adequate representation.

## 5. Conclusions

This paper provides an in-depth description of DUET—a theoretically grounded, dyadic-based lifestyle intervention for cancer prevention and control that is delivered and evaluated exclusively using remote technology. The report includes specific information on the overall protocol, intervention, and measures. Moreover, details are presented on the recruitment for this RCT which was successful in achieving its total accrual target with broad representation across the United States, and which also surpassed benchmarks for racial and ethnic representation.

## Figures and Tables

**Table 1:** DUET diet and exercise sessions.

Week	Topic/Brief Description
1	What Can You Do to Lower Your Risk of Cancer? American Institute of Cancer Research’s (AICR) dietary recommendations to lower cancer risk and why they are important.
2	Get on Track for Success! Using the Fitbit Aria weight scale each day and tracking tips and tools to promote weight loss.
3	Be Safe While Losing Weight! Importance of setting safe weekly weight loss goals that reduce the risk of sarcopenia.
4	Moving Towards Better Health Incremental goal setting to ultimately achieve 150 min of aerobic, resistance and flexibility exercises each week.
5	Let’s Get Physical and Step It Up! Use of the Fitbit wrist monitor to track physical activity and making a safe exercise plan with incremental goals
6	Be S.M.A.R.T. About Physical Activity and Exercise. How to make exercise goals specific, measurable, achievable, relevant and time-based.
7	The Sweet ‘n Low-down on Sugar and Fasting. Setting goals to limit of sugar intake and reviewing the concept and evidence for intermittent fasting.
8	Been Resisting “Resistance” Exercises? Importance of resistance exercise and instructions on how to perform them safely.
9	Yes, Portion Size Does Matter! Determining and tracking portions sizes; managing temptation while at the grocery store and dining out.
10	Why Are Bending Down and Touching Your Toes So Important for Good Health? How flexibility and balance exercises promote strength, and prevent falls and pain; instructions on how to begin safely.
11	Red and Processed Meats: How Can Something So Good Be So Bad? AICR recommendations on limiting red and processed meats. Harnessing social support to make dietary changes.
12	Did You Know that Your Surroundings Can Make You More Likely to Exercise? Managing environmental influences in support and promotion of good exercise habits.
13	Get the Skinny on Trimming the Fat. How high-fat foods contribute to risk of cancer and comorbidities; understanding different types of fat and food sources.
14	Reaping the Benefits of Whole Grains. Recommendations for whole grain daily intake and food sources.
15	Being Labeled is Not Always Bad. Importance of reading food labels and how.
16	Too pooped to Make Healthy Diet Choices? Recognizing and managing fatigue in support of healthy food preparation and making good choices.
17	Super Food Heroes: Fruits and Vegetables. Vegetables and fruits as sources of fiber, phytochemicals and antioxidants to reduce the risk for cancer and comorbidities; recommendation on sources, daily intake and serving sizes.
18	Problem Solving Strategies to Help You Get More Healthy Foods into Your Diet Identifying barriers/problems, brainstorming solutions, evaluating pros/cons, and developing an action plan.
19	Have Concerns About Pesticides Been Bugging You? Strategies to reduce pesticides in the diet and on foods; money and timesaving tips.
20	Want to Join the Party Without Blowing Your Diet? Recommendations on alcohol and cancer risk; making healthy choices when attending/hosting social gatherings.
21	Need a Break From Stress? Recognizing how stress influences physical and emotional wellbeing and strategies to manage it.
22	Why Am I Hungry All the Time? How to recognize hunger, and manage emotional or habitual eating.
23	Are Supplements Really Good for You? Information on safety and recommendations for supplement use
24	You did it! You Completed the DUET Program! Celebrating healthful eating and exercise behavior changes with positive rewards and planning for maintenance

**Table 2:** Outcome measures.

**PRIMARY OUTCOME**
**Body Weight:** Weight is measured in light clothing without shoes. Zoom^®^ images are captured of the “zeroed” scale display, the participant actively weighing, and final images of the display showing the participant’s weight. The assessor verifies the weight with both the participant and partner. Weight is measured twice, and the average taken for analyses.
**SECONDARY OUTCOMES**
**Waist circumference:** The participant faces the camera and positions clothing to reveal midriff; as the partner is coached to encircle the waist with one of the ribbons at the level of the umbilicus [18]. As the participant rotates, the assessor checks to assure the ribbon is flat against the skin and parallel to the floor. Upon exhale, the partner uses a felt-tip marker to mark the ribbon at the point of overlap. The process is repeated with the second ribbon. Both ribbons are returned to the study office and measured in centimeters and the average taken for analyses. **Diet Quality:** A trained nutritionist conducts telephone-based dietary recalls of a non-consecutive weekday and weekend day using the National Cancer Institute (NCI)-developed Automated Self-Administered 24-h (ASA24) recall dietary assessment web-based tool (https://epi.grants.cancer.gov/asa24/, accessed on 27 September 2021). Calorie intake and nutrient density are averaged over the 2 days for each time point and Diet Quality is calculated using the Healthy Eating Index (HEI)-2015 [19]. **Physical Activity:** Programmed actigraphs (Fort Walton, FL, USA) objectively capture physical activity over a 7-day period and are then downloaded and processed using procedures and software supplied by the manufacturer and using methods similar to those we have reported previously [20,21]. Physical activity also will be measured by self-report using the Godin Leisure-Time Exercise Questionnaire, given its excellent reliability and validity with cancer survivors [22,23]. **Physical Performance Testing:** The Senior Fitness Battery assesses physical performance objectively across multiple domains, is sensitive to change, minimizes ceiling effects, and has normative scores [24]. Usually conducted in-person, tests were adapted to virtual use, refined, and then evaluated for validity and reliability [17]; arm curls and grip strength, were omitted because of excessive equipment and postage costs. - 30-s chair stand (lower body strength): A standard 18” unpadded chair is used, though if the participant does not have one, the identical chair is used for both baseline and follow-up assessments. The participant sits in view of the camera and is instructed to cross arms with hands on shoulders. Upon the assessor’s signal to start, the participant stands up and sits down as many times as possible during a 30-s timed period. - 8′ Get Up & Go (agility, dynamic balance): Participant begins seated with crossed arms and hands on shoulders while the partner places a sticker and the end of the 8′cord (from mailed supplies) beneath the toe and extends the cord fully in front of the chair. The endpoint is marked by a soccer cone and the cord removed. The camera is positioned to capture the full course with a focus on the chair (start and end points for this test). Upon the signal to start, the participant stands, walks as fast as possible (without running) around the cone, returns to the chair, and sits down. The test is timed using the video—starting from the sign of movement until seated again. - 8′ Walk (gait speed): The chair is removed, and the participant stands with their toe on the sticker (see test above). Upon the signal to start, they walk as fast as possible through the 8′point marked by 2 soccer cones (another cone is added to increase visibility of the finish line). This test also is timed using the video, starting from the sign of movement until the finish line is crossed. - Sit-and-reach (flexibility): Seated on the edge of the chair, the participant extends one leg with their heel on the floor, the knee straightened, and the toe pointed to the ceiling. The camera captures the side view, and the assessor guides the participant to overlap their hands and extend them towards the toe. The partner measures the distance from the middle finger to the big toe with a vinyl tape measure. Positive values are recorded for over-reaching, negative for under-reaching, and zero for touching. - Back scratch (flexibility): The camera captures a back view while the participant reaches over their same shoulder while at the same time reaching their other arm directly back in an attempt to their clasp hands. The partner measures the distance between the closest fingers. Positive for over-reach, negative for under-reaching, zero for touching. - 2-min step test (endurance): The partner is instructed to palpate the participant to locate their iliac crest and then uses the vinyl tape measure to record the distance to the top of the patella, which is called-out to the assessor. The assessor calculates the midpoint, which is denoted by a sticker. Then the partner is asked to measure the distance from the sticker to the floor and call-out the value to the assessor. The assessor records this value for future testing and instructs the partner to measure this distance against a wall and to mark it with another sticker. The camera captures the side view and upon the command to start, the participant is instructed to “march in place” for 2 min making sure to bring their knees up to point of the sticker. The participant is instructed not to talk, and to take breaks, and briefly reach out to the wall to regain balance as needed while timer continues (partners are instructed to “spot” the participant as needed). The assessor counts steps during the 2-min period (steps not reaching the mark are not counted). **Balance Testing:** Zoom^®^ captures side-by-side, semi-tandem and tandem stance balance testing as advocated by the Centers for Disease Control [25]. To reduce ceiling effects, the latter test is extended for up to two minutes (or until the time the stance is broken). **Circulating Biomarkers:** DBS captured on the designated card are dried thoroughly (>4 h at room temperature), then inserted into a foil pouch with desiccant and frozen (0 F^o^ or below) until analyzed. DBS eluents are batch-tested against known standards for insulin, glucose, leptin, adiponectin, high density lipoprotein (HDL) and total cholesterol, triglycerides, interleukin-6 (IL6), c-reactive protein (CRP) and tumor necrosis factor alpha (TNFα) at the University of Washington as described previously [26]. Values are expressed in plasma equivalent terms. **Quality of Life:** The PROMIS global health scale and the EuroQOL-5D-5L (EQ-5D-5L) will be used to measure QOL [27]. The EQ-5D-5L includes 5 dimensions (Mobility, Self-care, Pain/Discomfort, and Anxiety/Depression) and the scores are used to calculate Quality Adjusted Life Years. **Comorbidity:** The Older Americans Resources & Services (OARS) Comorbidity Index (43-items) will assess the number and severity of chronic medical conditions and symptoms. Since falls are a particular issue in this population, an item validated by Chen & Janke that assesses falls in the past year also will be included [28,29].

**Table 3:** Study sample characteristics of DUET cancer survivors and partners *.

	Survivors (*n* = 56)	Partners (*n* = 56)	*p*-Value **
Cancer Diagnosis			<0.001
(*n*/%) *			
- Breast	45 (80%)	4 (7%)	
- Colorectal	1 (2%)	0	
- Gynecologic	2 (4%)	2 (4%)	
- Genitourinary	8 (14%)	1 (2%)	
Months elapsed since diagnosis			
- Mean (sd) (*n* = 53)	67.5 (72)		
- Range	10–303		
- Miles between Survivor and Partner (*n*/%)			
- 0 (cohabitate)	24 (43%)		
- Greater than 0, but less than 5	12 (21%)		
- 5 to 10	8 (14%)		
- More than 10	12 (21%)		
Race/Ethnicity			0.8
- Non-Hispanic White	35 (63%)	34 (61%)	
- Hispanic White	0	1 (2%)	
- Non-Hispanic Black	19 (34%)	21 (38%)	
- Hispanic Black	1 (2%)	0	
- Other	1 (2%)	0	
Gender (n/%)			0.03
- Male	8 (14%)	18 (32%)	
- Female	48 (86%)	38 (68%)	
Age (years)			0.1
- Mean (sd)	60.3 (11)	56.5 (14.3)	
- Range	32 79	23 81	
Educational Status			0.9
- High School Graduate	7 (13%)	9 (16%)	
- Some College/Junior College/Trade School	18 (32%)	17 (30%)	
- College Graduate/Post Graduate	30 (54%)	29 (52%)	
- Unknown	1 (2%)	1 (2%)	
Income			0.5
- Less than $50k/year	11 (20%)	7 (13%)	
- $50k/year or more	24 (43%)	23 (41%)	
- Unreported or Refused/Unknown	21 (38%)	26 (46%)	
Rural (*n*/%)	4 (7%)	5 (9%)	1
BMI (kg/m^2^) Mean (sd)	31.8 (5.8)	32.9 (6.1)	0.3
Employment (*n*/%)			1
- Employed	31 (55%)	31 (55%)	
- Retired	18 (32%)	18 (32%)	
- Other	7 (13%)	7 (13%)	

* Information on cancer-type was verified for cancer survivors, but was self-reported for supportive partners. ** For cancer diagnosis, participants with cancer were compared to participants without cancer; for race/ethnicity, only Non-Hispanic Whites and Non-Hispanic Blacks were compared; for educational status, participants with a response of unknown were excluded from the analysis.

## Data Availability

Not applicable.
