# Correction: Pekmezi et al. Rationale and Methods for a Randomized Controlled Trial of a Dyadic, Web-Based, Weight Loss Intervention among Cancer Survivors and Partners: The DUET Study. *Nutrients* 2021, *13*, 3472

**DOI:** 10.3390/nu14153141

**Published:** 2022-07-29

**Authors:** Dorothy W. Pekmezi, Tracy E. Crane, Robert A. Oster, Laura Q. Rogers, Teri Hoenemeyer, David Farrell, William W. Cole, Kathleen Wolin, Hoda Badr, Wendy Demark-Wahnefried

**Affiliations:** 1 Department of Health Behavior, University of Alabama at Birmingham (UAB), Birmingham, AL 35294, USA; 2 O’Neal Comprehensive Cancer Center at University of Alabama at Birmingham (UAB), Birmingham, AL 35294, USA; roster@uabmc.edu (R.A.O.); lqrogers@uabmc.edu (L.Q.R.); demark@uab.edu (W.D.-W.); 3 Sylvester Comprehensive Cancer Center, Miami, FL 33136, USA; tecrane@med.miami.edu; 4 Division of Preventive Medicine, Department of Medicine, Birmingham, AL 35294, USA; 5 Department of Nutrition Sciences, University of Alabama at Birmingham (UAB), Birmingham, AL 35294, USA; tgw318@uab.edu (T.H.); colew14@uab.edu (W.W.C.); 6 People Designs, Inc., Durham, NC 27705, USA; dfarrell@peopledesigns.com; 7 Coeus Health, LLC, Chicago, IL 60614, USA; kate@drkatewolin.com; 8 Department of Medicine, Epidemiology and Population Sciences, Baylor College of Medicine, Houston, TX 77030, USA; hoda.badr@bcm.edu

## Modifications to the Main Text and Table 2

The authors would like to correct errors in their prior publication [1]. In the original article, there were mistakes regarding the measures used for comorbidity and quality of life (Table 2) and depression (in the text, Materials and Methods, Section 2.5 Assessments). The correct measures were the Older Americans Resources & Services (OARS) Comorbidity Index [2,3], the EQ-5D-5L [4], and the Patient Reported Outcomes Measurement Information System (PROMIS) Global Health and Emotional Distress/Depression subscales [5], not the Charlson comorbidity index [6,7], RAND36 [8], and Center for Epidemiologic Studies of Depression [9].

The authors apologize for any inconvenience caused and state that the scientific conclusions are unaffected.
